# Iron metabolism: backfire of cancer cell stemness and therapeutic modalities

**DOI:** 10.1186/s12935-024-03329-x

**Published:** 2024-05-04

**Authors:** Rong Yu, Yinhui Hang, Hsiang-i Tsai, Dongqing Wang, Haitao Zhu

**Affiliations:** 1 Institute of Medical Imaging and Artificial Intelligence, Jiangsu University, Zhenjiang, 212001 China; 2 Department of Medical Imaging, The Affiliated Hospital of Jiangsu University, Zhenjiang, 212001 China

**Keywords:** Cancer stem cells (CSCs), Ferroptosis, Iron metabolism, Nanotechnology, Photodynamic diagnosis/Photodynamic therapy, Stemness

## Abstract

Cancer stem cells (CSCs), with their ability of self-renewal, unlimited proliferation, and multi-directional differentiation, contribute to tumorigenesis, metastasis, recurrence, and resistance to conventional therapy and immunotherapy. Eliminating CSCs has long been thought to prevent tumorigenesis. Although known to negatively impact tumor prognosis, research revealed the unexpected role of iron metabolism as a key regulator of CSCs. This review explores recent advances in iron metabolism in CSCs, conventional cancer therapies targeting iron biochemistry, therapeutic resistance in these cells, and potential treatment options that could overcome them. These findings provide important insights into therapeutic modalities against intractable cancers.

## Introduction

Although a wide range of anticancer treatments have emerged, from established interventions like surgery, radiotherapy, and chemotherapy to newer approaches like neoadjuvant therapy, targeted therapy, and immunotherapy, the global burden of cancer remains substantial. Notably, cancer persists as the second leading cause of death worldwide, emphasizing the need for further therapeutic advancements [1]. Meanwhile, numerous reports have demonstrated that cancer treatment failure is attributed to the presence of cancer stem cells (CSCs) [2, 3].

CSCs were first detected in leukemia and later identified in various solid tumors such as breast, prostate, lung, pancreatic, and colorectal cancers, among others [4]. CSCs comprise a rare cell subpopulation with stem-like properties, including self-renewal, unlimited proliferation, multidirectional differentiation, invasion, and metastasis. As CSCs are rare and have few known specific biomarkers, their elimination remains challenging. The therapeutic resistance of CSCs can be attributed to their unique intrinsic cell molecular biology and extrinsic tumor microenvironment (TME). Moreover, metabolic reprogramming in CSCs and its influence on their fate determination is attracting growing attention. Unlike non-CSCs and parental cancer cells, CSCs rely on their unique metabolic pathways for survival. It is now understood that glucose, amino acid, and fatty acid metabolism are all crucial for the survival of CSCs.

Interestingly, the metabolism of trace elements also plays a role in various biological processes of CSCs. Zinc has been demonstrated to potentiate the tumorigenic capacity of CSCs. This process is tightly regulated by a network of proteins involved in cellular zinc homeostasis, with the SLC39 (Zrt- and Irt-like proteins/ZIP) family playing a key role in zinc influx. Notably, ZIP4, a recently identified marker for ovarian CSCs [5], has been reported to exhibit elevated expression in pancreatic cancer as well. Furthermore, studies suggest that ZIP4 promotes CSCs stemness, including enhanced invasion, metastasis, and resistance to chemotherapy [6]. In addition, it has been reported that the high level of zinc in breast CSCs directly enhances tumorigenicity [7]. Manganese (Mn), an essential dietary element for intracellular activities, can participate in the synthesis of Mn superoxide dismutase (MnSOD) as a cofactor, enhancing cell migration, invasion, sphere formation, and colony formation, and improving the expression of stem cell markers in tongue squamous cell carcinoma [8, 9]. Calcium has also been reported to play a role in the biological processes of CSCs. Calcium channels on the plasma membrane, including ligand-gated channels (LGC), store-operated channels (SOC), and voltage-operated channels (VOC), positively correlate with stemness [10]. Recent studies have highlighted the significant role of iron metabolism in CSCs, suggesting it is a promising new target for cancer treatment [11, 12]. This review summarizes how iron metabolism contributes to CSCs resistance and explores novel therapeutic approaches to target these cells.

## Cancer stem cells

### Biological characteristics

**Fig. 1:**
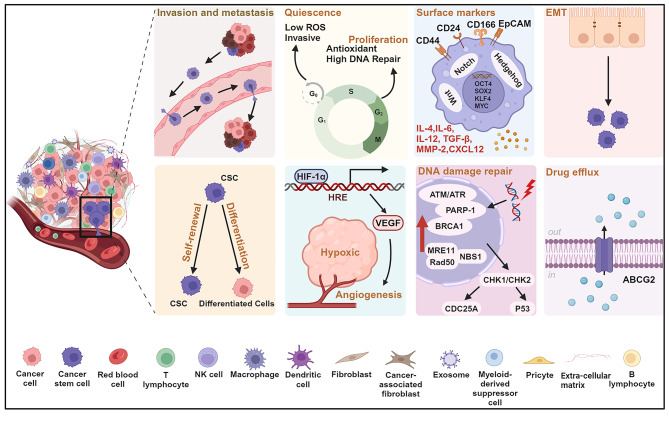
Biological characteristics that endue cancer stem cells (CSCs) with therapeutic resistance. CSCs express tissue-specific surface markers such as CD44, CD166, CD24, epithelial cell adhesion molecule (EpCAM), and others and can self-renew and differentiate into heterogeneous subpopulations. These properties enable CSCs to contribute to tumor recurrence and metastasis. In the G0 phase, CSCs enter a quiescent state in which they are insensitive to the adverse effects of therapeutic stress and the microenvironmental cues and can maintain their stemness. ABCG2 upregulation promotes multidrug resistance (MDR), and the enhanced DNA damage response (DDR) promotes radiation resistance by upregulating proteins involved in DNA repair such as ataxia telangiectasia mutated (ATM), ataxia telangiectasia and Rad3-related (ATR).They are the key sensors of DNA damage, which in turn form complexes with poly (ADP-ribose) polymerase 1 (PARP-1), breast cancer susceptibility gene 1 (BRCA1) to phosphorylate checkpoint kinases CHK1/CHK2 and drive the activation of targeted proteins, including CDC25 family member A (CDC25A), p53, etc. The MRE11-RAD50-NBS1 (MRN) complex, a double-strand break sensor, is engaged in various DNA damage repair pathways. Hypoxic microenvironment promotes angiogenesis by facilitating the binding of hypoxia-inducible factor-1 alpha (HIF-1α) and hypoxia response element (HRE) to induce vascular endothelial growth factor (VEGF) release. CSCs and their surrounding cells secrete various chemokines, cytokines, and growth factors including transforming growth factor-beta (TGF-β), matrix metalloproteinase 2 (MMP2), C-X-C motif chemokine 12 (CXCL12), and others, thereby maintaining the homeostasis of the CSCs niche. Epithelial-mesenchymal transition (EMT) activates stem cell-related signaling pathways and enhances stemness. **Abbreviations**: ROS, reactive oxygen species; OCT4, octamer-binding transcription factor 4; SOX2, SRY (sex-determining region Y)-box 2; KLF4, Kruppel-like factor 4; MYC, myelocytomatosis; ABCG2, ATP-binding cassette sub-family G member 2

Cancer stem cells constitute a special tumor cell subpopulation that drives tumor initiation, metastasis, and recurrence [13]. However, they account for only 0.01–2% of the total tumor volume [4]. While various surface markers have been reported for separation and identification of CSCs, including CD44, CD24, CD166, aldehyde dehydrogenase (ALDH), and epithelial cell adhesion molecule (EpCAM) [14], it is the unique combination of their biological properties that presents a major obstacle to effective therapy (Fig. 1).

#### Self-renewal and unlimited proliferation

CSCs possess the unique capability of self-renewal through both symmetrical and asymmetrical cell division. The latter involves generating two daughter cells: one that retains the ability to self-renew and another that undergoes differentiation. Symmetrical division, on the other hand, produces two identical daughter cells with unlimited proliferative potential [15]. This combination of self-renewal mechanisms and indefinite proliferation enables CSCs to drive tumorigenesis and maintain tumor mass [16]. Lapidot et al. reported that stem cells isolated from patients with acute myelogenous leukemia (AML) could form new tumors in severe combined immunodeficiency (SCID) mice [4].

#### Multidirectional differentiation

CSCs have been described as a class of pluripotent cancer cells that reside at the apex of the hierarchical tree. These cells possess the ability to differentiate into non-stem cancer cells, leading to phenotypic and functional heterogeneity within the tumor. CSCs-derived tumor xenografts exhibit marked cytological diversity, highlighting how their multidirectional differentiation contributes to both tumorigenesis and cancer progression [17, 18].

#### Invasion and metastasis

Metastasis, a multi-step process involving invasion, intravasation, dissemination through circulation, extravasation, reactivation, and colonization, presents a major challenge in cancer management [19]. Unlike primary tumors that are often localized and treatable with surgery and/or radiation, metastasis is systemic and spreads throughout the body. Only metastatic stem cells (MetSCs) survive this rigorous process and are capable of initiating new tumor formation, infiltrating host tissues, and further metastasis [20]. Pang et al. demonstrated that isolated CD26^+^ CSCs exhibit enhanced abilities for metastasis, invasion, and resistance to chemotherapy, ultimately leading to distant liver metastasis [21].

#### Other properties

CSCs exhibit robust chemotherapy and radiotherapy resistance due to several factors, including strong ATP-binding cassette (ABC) transporter-mediated multidrug resistance (MDR), abundant endogenous antioxidant and detoxification pathways, upregulation of anti-apoptotic proteins, enhanced DNA damage repair capability (DDR), and reversible quiescence [22–28].

### Maintenance of CSCs stemness

CSCs are thought to be the seeds of certain malignant tumor behaviors. CSCs are regulated by a complex, interwoven network of intracellular transcription factors (TFs) and signaling pathways as well as extracellular factors, such as vascular niches, hypoxia, cancer-associated fibroblasts, the extracellular matrix (ECM), and exosomes. Metabolic reprogramming has also been implicated in maintaining CSCs stemness [29, 30].

#### TFs and signaling pathways

CSCs stemness is mainly regulated by Yamanaka factors, namely, octamer-binding transcription factor 4 (OCT4), SRY (sex-determining region Y)-box 2 (SOX2), Kruppel-like factor 4 (KLF4), and myelocytomatosis (MYC). Collectively, these are designated as OSKM [31]. It has been established that these factors, individually or in combination, regulate CSCs self-renewal and differentiation [32, 33]. Lu et al. reported that Oct4 and Nanog knockdown could attenuate the stemness of pancreatic CSCs by downregulating the migration-related gene CXC motif chemokine receptor 4 (CXCR4) and the invasion-related genes matrix metalloproteinase 2 (MMP2), matrix metalloproteinase 9 (MMP9), and tissue inhibitor of metalloproteinase (TIMP1). Oct4 and Nanog knockdown also enhanced pancreatic CSCs chemosensitivity by downregulating ATP-binding cassette superfamily G member 2 (ABCG2) [34].

Intracellular signaling pathways involved in maintaining CSC stemness include nuclear factor-κB (NF-κB), Wnt/β-catenin, Notch, Janus kinase/signal transducer and activator of transcription (JAK/STAT), Hedgehog (Hh), phosphoinositide 3-kinase/AKT/mammalian target of rapamycin (PI3K/AKT/mTOR), transforming growth factor-β (TGF-β)/SMAD, and peroxisome proliferator-activated receptor (PPAR) [35]. Giancotti reported that the JAK/STAT3 pathway works synergistically with Wnt/β-catenin and Notch to induce the stemness TFs SOX2, OCT4, and NANOG and, by extension, promote CSCs self-renewal and metastasis [36].

#### Microenvironment

CSCs reside in a specific microenvironment called the CSCs niche [37]. This niche comprises the extracellular matrix, cytokines, chemokines, growth factors, various types of cells surrounding the CSCs (fibroblasts, immunocytes, macrophages, etc.), and acellular components like exosomes. It promotes stemness and shields CSCs from chemotherapy, immunotherapy, and radiotherapy [26]. Growing evidence suggests that CSCs tend to localize to hypoxic and perivascular niches [38, 39].

In glioblastoma (GBM), hypoxia enhances CSCs-mediated immunosuppression by activating the STAT3 pathway and hypoxia-inducible factor-1 alpha (HIF-1α) and hindering T-cell activation [40]. In mucoepidermoid carcinoma, it promotes stemness and maintains the mesenchymal state by activating the TGF-β and Wnt/β-catenin pathways [41]. Meanwhile, in triple-negative breast cancer (TNBC) CSCs, it upregulates stemness-related transcription factors (c-Myc, Oct4, Klf4, and Sox2) and enhances mammosphere formation [42]. Notably, hypoxia also stimulates angiogenesis by inducing angiogenic factors like vascular endothelial growth factor (VEGF) [43].

Beck et al. reported that VEGF stimulates angiogenesis in a paracrine manner, creating a perivascular niche that enhances the self-renewal and differentiation potential of CSCs derived from skin squamous cell carcinoma [44]. Additionally, VEGF induces epithelial-mesenchymal transition (EMT) in head and neck cancer cells, promoting their migration and tumorigenicity while enhancing their resistance to anoikis [45]. These findings suggest that CSCs, like those in hematological and brain malignancies, might reside in vascular niches [46, 47].

#### Metabolism

Cellular metabolism is not a passive player in stem cell lineage commitment but actively determines their fate. Metabolic reprogramming and stemness are closely linked, with glucose metabolism being the most common alteration in stem cells [48]. While bulk tumor cells rely solely on glycolysis, CSCs exhibit unique metabolic flexibility, utilizing both glycolysis and oxidative phosphorylation (OXPHOS) depending on the context [49, 50]. Glycolysis is the preferred pathway for CSCs in nasopharyngeal, hepatocellular, breast, lung, and colorectal cancers. Upregulation of glycolytic enzymes like glucose transporter 1 (GLUT1), hexokinase isoform II (HK II), and aldehyde dehydrogenase (ALDH) enhances self-renewal, tumor initiation, metastasis, and chemo/radiotherapy resistance in these CSCs [51–54]. However, current evidence suggests that CSCs in some cancer lines are OXPHOS-dependent [55, 56]. TFs like peroxisome proliferator-activated receptor-γ coactivator 1-alpha (PGC-1α), the proto-oncogene MYC, and the anti-apoptotic protein Mcl-1 regulate mitochondrial biogenesis and OXPHOS, maintaining breast CSCs stemness by inducing stemness markers and mammosphere formation [57, 58]. Additionally, oncogene ablation-resistant pancreatic cancer cells with CSCs-like traits rely on OXPHOS for survival [59]. Lipid metabolism also regulates the ability of self-renewal, metastasis, and tumor initiation by providing bioenergy through fatty acid oxidation and activating stem cell-related signaling pathways [60–65]. Additionally, increased metabolism of specific amino acids (e.g., glutamine, lysine, serine, and branched-chain amino acids) plays an important role in maintaining CSCs stemness [66–69].

However, effective therapies to eradicate cancer stem cells remain elusive. Recent research has focused on the mechanisms of how iron metabolism is reprogrammed in CSCs, with the aim of identifying potential targets for the development of efficacious anticancer therapies.

## Iron metabolism

### Iron metabolism in normal cells

Understanding iron metabolism within cancer stem cells requires a foundational knowledge of how this metabolic process functions in normal cells. Iron, an essential trace element in humans, plays critical roles in oxygen and electron transport, DNA synthesis and repair [70]. However, free iron can catalyze the Fenton reaction, generating harmful reactive oxygen species (ROS) upon interaction with hydrogen peroxide (H_2_O_2_). To mitigate oxidative stress and DNA damage, normal cells meticulously maintain iron homeostasis through a well-defined system encompassing uptake, redistribution, efflux, and regulation [71].

#### Iron uptake

Iron exists in two redox states, namely, oxidized ferric iron (Fe^3+^) and reduced ferrous iron (Fe^2+^). In a standard diet, plant-derived inorganic iron (Fe^3+^) accounts for 80–90%, while the remaining 10% is heme iron (Fe^2+^) associated with meat intake [7]. Ingested inorganic iron must first be reduced to its ferrous form through ferrireductases, particularly duodenal cytochrome b reductase (Dcytb), which may involve other ferrireductases, then be transported into enterocytes by divalent metal transporter 1 (DMT1). Within enterocytes, iron is transiently collected in the labile iron pool (LIP) and then carried by chaperones such as Poly(rC)-binding proteins (PCBPs) to locations for storage in ferritin, or exported into the circulation by ferroportin (FPN). In plasma, Fe^2+^ is oxidized to Fe^3+^ by the ferroxidase hephaestin (HEPH) [52]. Transferrin (Tf), a glycoprotein with two high-affinity sites for ferric iron, binds to transferrin receptor 1 (TFR1) for receptor-mediated endocytosis (see ‘Iron trafficking and regulatory mechanisms in CSCs’).

Although most circulating iron is delivered to normal cells by the transferrin endocytic cycle, non-transferrin-bound iron (NTBI) occurs when the plasma iron level exceeds the iron buffering capacity of transferrin. NTBI is mainly transported into cells by the solute carrier SLC39A14 (also called ZIP14) [72] and its paralogue SLC39A8 (also called ZIP8) [73]. Besides, cells can acquire iron bound to hyaluronic acid through the receptor CD44 and iron bound to siderophore-lipocalin 2 (LCN2) complex through the megalin–cubilin endocytic receptors or solute carrier family 22 member 17(SLC22A17) pathways [74, 75]. Ferritin present in small amounts within serum and other extracellular fluids represents an alternative iron source for cells. Scavenger receptor class A member 5 (SCARA5), T cell immunoglobulin and mucin domain-containing protein 2 (TIM-2), and transferrin receptor 1 (TFR1) can all mediate the endocytosis of this ferritin [76]. Additionally, heme iron acquisition occurs when macrophages engulf and degrade aged or damaged erythrocytes within phagolysosomes. This releases heme, which is then transported out of the phagosome into the cytoplasm by HRG1 (also known as SLC48A1). Finally, heme oxygenase 1 (HMOX1) catabolizes heme within the cytoplasm, liberating iron ions [77].

#### Iron redistribution

Following endocytosis of iron-containing cargoes or degradation through endolysosomes, iron enters the cytosolic labile iron pool (LIP) for redistribution. This redistribution involves two main pathways: storage or utilization. Iron can be stored in ferritin, either in the cytosol or within mitochondria (mitochondrial ferritin, FTMT). Alternatively, it can be transferred into the mitochondrial matrix for essential functions like iron-sulfur (Fe-S) cluster assembly and heme biosynthesis. This transfer is facilitated by divalent metal transporter 1 (DMT1) on the outer mitochondrial membrane and mitoferrins 1 and 2 (MFRN1 and MFRN2) on the inner membrane [78].

Fe-S cluster biogenesis is an evolutionarily conserved and intricate process involving multiple proteins, including the ISC core complex (NFS1/ISD11/acyl carrier protein (ACP)/ISCU). De novo assembly begins with the desulfurase NFS1 converting cysteine to alanine. This releases sulfur to the scaffold protein ISCU, forming a [2Fe-2S] cluster through a conformational change, potentially activated by frataxin (FXN) [79]. The accessory protein ISD11 stabilizes the key enzyme NFS1 in the ISC core complex, ensuring efficient Fe-S cluster biogenesis. Interestingly, the mitochondrial acyl-carrier protein NDUFAB1 also binds ISD11 within the complex, suggesting potential regulatory roles [80]. Newly synthesized [2Fe-2 S] clusters have two fates: they can be directly incorporated into target proteins or undergo further transformation into [4Fe-4S] clusters. Intriguingly, two models explain cytoplasmic Fe-S biogenesis. One proposes that extramitochondrial Fe-S proteins rely on the mitochondrial ISC machinery to synthesize a sulfur-containing intermediate (X-S) exported to the cytoplasm by the ABC transporter ABCB7 for [4Fe-4S] clusters assembly. Alternatively, the other model suggests independent de novo synthesis within the cytosol, independent of mitochondrial involvement [81]. Heme synthesis is a multistep process involving reactions inside and outside the mitochondria. It begins with the combination of glycine and succinyl-Coenzyme A (succinyl-CoA) to generate 5-aminolevulinate (ALA) and terminates with iron insertion into the protoporphyrin (PPIX) rings by ferrochelatase (FECH).

#### Iron efflux

Besides the most common ferrous iron exporter, ferroportin (FPN), other iron export pathways exist. Nuclear receptor co-activator 4 (NCOA4)-bound ferritin can be released into the extracellular space through either secretory autophagy or endosomal microautophagy. Additionally, heme iron can be exported via the transporter Feline Leukemia Virus Subgroup C Receptor 1a (FLVCR1a) or other transporters like ABCG2 and ATP-binding cassette subfamily C member 5 (ABCC5) [82].

#### Regulation of iron homeostasis

The iron-responsive element/iron-regulatory protein (IRE/IRP) system monitors cellular iron homeostasis through posttranscriptional regulation. The key players in this network are iron-regulatory protein 1 (IRP1, encoded by aconitase ACO1) and iron-regulatory protein 2 (IRP2, encoded by iron-responsive element binding protein 2 (IREB2)). They bind to iron-responsive elements (IREs) located in the mRNAs of various critical proteins involved in iron metabolism. These IREs, forming unique stem-loop structures in the 5’ or 3’ untranslated regions (UTRs) of mRNA transcripts, control target mRNA translation [77]. Depending on cellular iron levels, IRPs fine-tune the expression of proteins implicated in iron import (TFR1, DMT1), storage (ferritin H and L subunits), and efflux (FPN). This maintains optimal intracellular iron concentration and protects cells from ferrotoxicity [83, 84]. When iron is abundant, it binds to IRPs, triggering a conformational change. This disrupts the IRP-IRE interaction in the 5’-UTR, enabling ferritin and FPN biosynthesis, while promoting TfR1 mRNA degradation at the 3’-UTR. When cells are iron deficient, the IRPs bind the 5′ IREs in the FPN and ferritin mRNAs, inhibiting their translation, and bind the 3′ IREs in the TfR1 mRNA to prevent transcripts from endonuclease degradation. In this manner, the mRNA half-life is prolonged, and translation is promoted. Additionally, IRP2 is regulated by the ubiquitin ligase leucine-rich repeat 5 F-box protein (FBXL5), which recruits IRP2 for ubiquitination and degradation by the proteasome when iron is abundant. When iron is deficient, FBXL5 is polyubiquitinated via the E3 ubiquitin-protein ligase HECT domain and RCC1-like domain 2 (HERC2), leading to its degradation, and IRP2 accumulation [85, 86].

Alternatively, nuclear receptor coactivator 4 (NCOA4) can sense changes in iron content. When iron is deficient, NCOA4 mediates autophagy to degrade ferritin (ferritinophagy), releasing iron back into the labile iron pool. Conversely, when iron is abundant, NCOA4 is blocked from binding ferritin and instead interacts with HERC2, leading to NCOA4’s own degradation by the proteasome, thus allowing ferritin accumulation. Furthermore, the Fenton chemistry, involving iron, poses a constant threat to cellular redox balance. To protect themselves, cells employ various antioxidant measures. The transcription factor nuclear factor erythroid 2-related factor 2 (Nrf2), activated by oxidative stress, plays a crucial role in regulating these defense mechanisms. Under stress, Nrf2 translocates to the nucleus and binds to the small musculoaponeurotic fibrosarcoma oncogene homologue (sMAF), activating antioxidant response elements (AREs) in the genes of various iron-related processes, including heme oxygenase-1 (HMOX1), SLC40A1, FECH, etc [87, 88]. Additionally, the prolyl hydroxylase domain (PHD)-hypoxia-inducible factor (HIF) axis regulates iron metabolism by influencing dietary iron absorption, erythropoietin (EPO) production, and hepcidin expression.

### Iron trafficking and regulatory mechanisms in CSCs

**Fig. 2:**
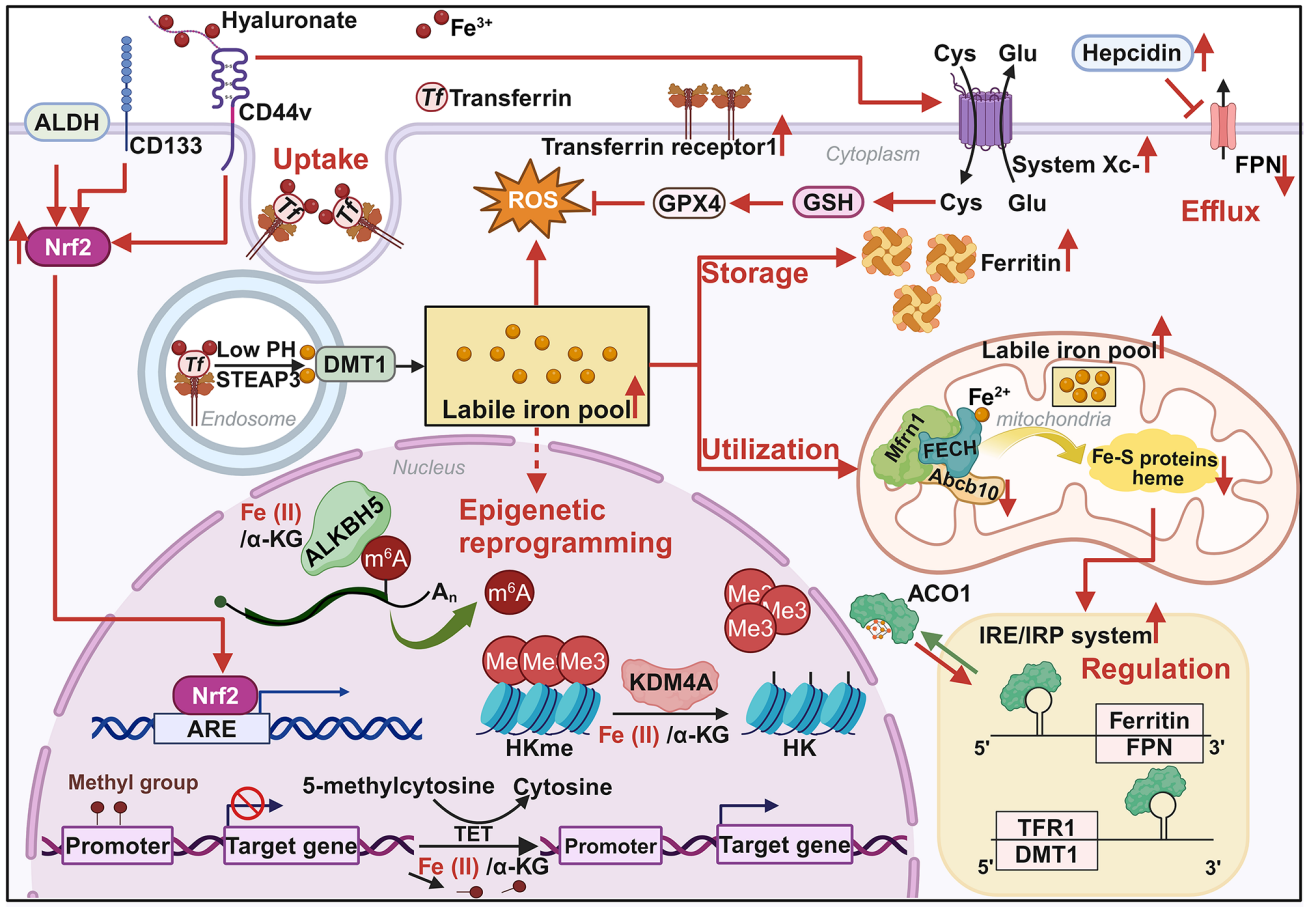
Iron metabolism in CSCs compared to non-CSCs. CSCs express proteins implicated in iron trafficking and are distinguished from non-CSCs (red arrows). CSCs promote ROS generation and regulate epigenetic reprogramming to maintain stemness by accumulating intracellular iron.The innate CD44/hyaluronate (Hyal) pathway promotes iron endocytosis while the upregulation of the xCT/GSH/GPX4 pathway and NRF2 renders CSCs resistant to oxidative damage. **Abbreviations**: CD44v, CD44 variant isoform; TF, transferrin; GPX4, glutathione peroxidase 4; GSH, glutathione; ALDH, aldehyde dehydrogenase; Cys, cystine; Glu, glutamate; DMT1, divalent metal transporter 1; FPN, ferroportin; GPX4, glutathione peroxidase 4; IRE/IRP system, iron-responsive element/iron regulatory protein system; NRF2, nuclear factor erythroid 2-related factor 2; ROS, reactive oxygen species; System Xc-, cystine/glutamic acid transporter; STEAP3, six epithelial transmembrane antigens of the prostate 3; Mfrn1, mitoferrin-1; FECH, ferrochelatase; Abcb10, ATP-binding cassette subfamily B member 10; TFR1, transferrin receptor 1; ARE, antioxidant response element; TET, The ten-eleven translocation proteins; ALKBH5, alkylation protein AlkB homolog 5; KDM4A, Lysine-specific demethylase 4 A

Intracellular iron accumulation, known as iron addiction, is a metabolic hallmark of CSCs [12, 89–91]. Understanding iron trafficking and regulatory mechanisms in CSCs is crucial for developing effective targeted anticancer therapies (Fig. 2).

Physiologically, most circulating iron binds to transferrin and its receptor TFR1, triggering membrane invagination and endosome formation. The declining pH within the endosome prompts Tf to release Fe^3+^, which is then reduced to Fe^2+^ by six-transmembrane epithelial antigen of prostate 3 (STEAP3). Subsequently, DMT1 translocates Fe^2+^ from the endosome to the cytosol [92, 93]. Xiao et al. reported the elevated TFR1 protein in CSCs derived from hepatocellular carcinoma. Notably, TFR1 knockdown or iron chelation not only downregulated stemness markers CD44 and CD133 but also reduced G1/G0 phase cells and repressed colony formation [94]. Interestingly, CD133 knockdown significantly enhanced Tf/TFR1 endocytosis, suggesting an iron-dependent regulation [95]. The transmembrane glycoprotein CD44, a recognized CSCs marker, mediates hyaluronate-dependent iron endocytosis, with its transcription regulated by iron-catalyzed histone demethylation. Therefore, the CD44/hyaluronate (Hyal) pathway represents a potent alternative for maintaining iron homeostasis in CSCs [96]. Collectively, these findings highlight how enhanced iron uptake contributes to CSCs expansion and stemness maintenance.

The protein ATP-binding cassette subfamily B member 10 (ABCB10) resides within the inner mitochondrial membrane and forms a complex with two other proteins, FECH and Mfrn1. This complex functions synergistically to incorporate iron into protoporphyrin IX (PPIX) during heme biosynthesis and also participates in the biogenesis of iron-sulfur (Fe-S) clusters within the mitochondria [97–100]. Interestingly, studies have shown that in breast and prostate cancers, ABCB10 protein levels are reduced through post-transcriptional mechanisms. This downregulation leads to an accumulation of iron within the mitochondria, as well as enhanced stemness and increased differentiation characteristics in cancer stem cells [101, 102].

The cytoplasmic iron storage protein ferritin sequesters intracellular iron in a non-toxic form. Composed of 24 subunits, including ferritin heavy chain (FHC) and ferritin light chain (FTL) subtypes, it maintains cellular redox homeostasis [103]. 3D spheres (SPHs) derived from cholangiocarcinoma and glioblastoma stem cells showed upregulation of ferritin protein, suggesting increased iron demand and dependence. Ferritin may enable CSCs to contend with excess free iron and its associated oxidative stress by sequestering iron ions. Additionally, it may regulate cell cycle progression through the STAT3-FOXM1 axis and serve as a negative prognostic factor for GBM [104, 105]. Meanwhile, the labile iron pool reacts with H_2_O_2_ through Fenton chemistry, generating highly reactive hydroxyl radicals (OH•). This reaction contributes to the regulation of lung CSCs phenotypes, including enhancing spheroid formation, increasing invasiveness, and upregulating the stemness biomarker ABCG2 [106]. Importantly, an increase in intracellular iron content promotes the metastasis of ovarian CSCs by inducing interleukin-6 (IL-6) biosynthesis and facilitating STAT3 phosphorylation and nuclear translocation [107–109].

Ferroportin, the main route for ferrous iron export, is negatively regulated by the peptide hormone hepcidin (HAMP). It has been established that HAMP induces internalization and degradation of FPN, thereby inhibiting Fe^2+^ transport [110, 111]. Basuli et al. reported an iron-retention profile in ovarian CSCs where FPN was downregulated while TFR1 was upregulated, both regulated by c-Myc. This suggested that ovarian CSCs were sensitive to iron chelation. Using a conditional doxycycline-driven promoter (FPN-tet-on), they upregulated FPN and significantly reduced tumor number, mass, and metastatic area [107]. Similarly, Wang et al. observed HAMP upregulation and FPN downregulation in ovarian CSCs, with both factors synergistically increasing intracellular iron levels. Exposure to the iron chelator deferoxamine (DFO) significantly reduced the stemness-related transcription factors Nanog and Sox2, mammosphere formation, and the CD44^+^/CD133^+^ and ALDH^+^ SK-OV-3 ovarian cancer cell subpopulations [112].

Cellular iron homeostasis is primarily maintained by the IRE/IRP regulatory network. IRPs bind to iron response elements (IREs) located in the untranslated regions (UTRs) of mRNAs, thereby regulating iron uptake, storage, utilization, and efflux. Rychtarcikova et al. reported that reduced cytosolic iron-sulfur (Fe-S) clusters activated the IRE/IRP system, leading to upregulated ACO1 expression, increased iron uptake, and decreased iron storage in breast and prostate CSCs [101]. Conversely, IRP activity decreases in the presence of sufficient metabolically available iron [104]. These findings suggest that CSCs are characterized by high intracellular iron loading and dependence. Moreover, NRF2 and its target genes were highly expressed in CD44^+^/CD24^−^ breast cancer cells, ALDH^+^ ovarian cancer cells, and CD133^+^ colon cancer cells [113]. NRF2 not only promotes the expression of antioxidant proteins such as forkhead box protein O3 (FOXO3), but also maintains the stemness of cancer cells by upregulating transcription factors such as Notch, Hedgehog, and β-catenin.

### Iron metabolism and EMT in CSCs

Cancer cell plasticity allows cells to undergo reversible shifts between distinct states, gaining new features. Epithelial-mesenchymal plasticity (EMP), including both epithelial-mesenchymal transition (EMT) and mesenchymal-epithelial transition (MET), contributes to CSCs generation and maintenance, tumor initiation, progression, metastasis, and therapeutic resistance [114–116]. EMT is a complex but coordinated cellular program where epithelial cells lose their polarity and detach, acquire mesenchymal-like phenotypes, become invasive, and migrate through the ECM [117, 118]. This process is regulated by various factors, including extracellular stimuli like cytokines, inflammation, and hypoxia, cell adhesion through integrins, and intracellular signaling pathways such as Notch, Hedgehog, Wnt/β-catenin, and transcription factors like SNAIL, TWIST, and ZEB.

Through the aforementioned regulatory networks, EMT can generate cells exhibiting stemness-like properties. Mani et al. discovered that ectopic Twist or Snail expression, or exposure to TGF-β1, activated EMT, driving epithelial carcinoma cells into a mesenchymal state and endowing them with CSCs-like properties such as increased expression of stemness markers, in vitro sphere formation ability, and broad metastasis in vivo [119, 120]. Simultaneously, EMT programs might regulate cancer cell self-renewal by controlling cell division modes, protect genome integrity, and facilitate DNA repair, which could explain why certain cancer cells with stem cell-like properties display high genomic stability [121, 122]. Additionally, EMT could either modulate microenvironmental niche interactions or activate the antioxidant response, both of which are involved in CSCs stemness maintenance [123–125].

Meanwhile, there is an increasing consensus suggesting that cellular iron is implicated in the regulation of EMT programs and stemness. Chen et al. demonstrated that the iron chelators di-2-pyridylketone-4,4,-dimethyl-3-thiosemicarbazone (Dp44mT) and Deferoxamine (DFO) attenuated TGF-β-induced EMT by maintaining the N-myc downstream-regulated gene 1 (Ndrg1)-mediated membrane localization of E-cadherin and β-catenin, thereby inhibiting EMT and promoting cellular adhesion [126]. Ndrg1 strongly suppresses metastasis during EMT. Ndrg1 expression increased when cancer cells were incubated with an iron chelator, but decreased when cultured with the iron donor ferric ammonium citrate (FAC) [127, 128]. This downregulation promoted characteristics of stemness, as evidenced by increased expression of cancer stem cell markers, enhanced colony formation, and increased invasiveness [129, 130]. In addition, FHC has emerged as a novel modulator in TGF-β1-induced EMT. By repressing FHC biosynthesis and increasing intracellular levels of LIP and ROS, TGF-β1 activates p38 mitogen-activated protein kinase (MAPK), promoting EMT and enhancing cancer cell stemness, evident by increased invasiveness and migration. This phenomenon has been observed in both acute myeloid leukemia and non-small-cell lung carcinoma [131]. Raggi et al. observed that iron supplementation could upregulate the genes involved in cancer cell stemness, including CD133, EpCAM, OCT4, cMYC, and others, and induce EMT-related factors such as β-catenin, ZEB1, SLUG, and SNAI2, while iron chelation reversed these effects [104]. These findings collectively suggest a strong link between iron metabolism and the modulation of EMT and stemness in cancer.

### Iron metabolism and epigenetic reprogramming in CSCs

Asymmetric cell division, a reversible inheritance process where daughter cells exhibit distinct fates, serves as the cornerstone for the generation of cancer stem cells. Epigenetic modifications, such as DNA methylation, histone modifications, chromatin remodeling, and RNA modification, regulate the genes implicated in CSCs maintenance and tumorigenesis [132]. Iron acts as an essential cofactor for epigenetic enzymes, linking the epigenetic machinery with cellular iron metabolism, which contributes to the regulation of stemness through processes like histone modifications and DNA and RNA demethylation [11].

Ten-eleven translocation (TET) and Jumonji C (JmjC) domain-containing families have been identified as demethylases that can remove methyl groups from DNA and histones, respectively. They are both depend upon Fe (II) and α-ketoglutarate [133–136]. Elevated levels of LIP and FTL stimulate TET protein-dependent oxidation of 5-methylcytosine (5mC) to 5-hydroxymethylcytosine (5hmC), consequently activating DNA demethylation. Conversely, treatment with DFO downregulates TET protein expression in breast cancer cells [137–139]. Prasad et al. demonstrated that in glioblastoma stem cells, hypoxia increased TET1 and TET3 expression, strengthening their binding to the promoter regions of the pluripotency genes OCT4 and NANOG, leading to both increased neurosphere formation and therapeutic resistance [140]. Similarly, hypoxia-mediated TET2 and TET3 upregulation promoted CpG island demethylation and induced Wilms’ tumor-1 (WT-1) protein expression in acute myeloid leukemia stem cells, which played an important role in regulating stem cell proliferation and differentiation [141]. TET1 knockdown mitigated hypoxia-induced EMT by downregulating mesenchymal genes including vimentin and N-cadherin and decreased migration and invasion activity in lung cancer cells [142]. Additionally, TET proteins play a crucial role in maintaining genomic integrity, as evidenced by the gradual accumulation of DNA damage in their absence (Tet2 and Tet3) [143]. It has been established that Lysine-specific demethylase 4 A (KDM4A or JMJD2A), part of the JmjC domain family of histone demethylases, specifically demethylates histone H3 lysine 9 trimethylation (H3K9me3) [144]. It is downregulated in response to iron chelation [145, 146]. Metzger et al. showed that KDM4A upregulation demethylated EGFR by downregulating H3K9me3 at the EGFR promoter, which increased the proliferation, sphere-forming capacity in vitro, and xenograft tumor growth of triple-negative breast CSCs in vivo [147].

An increasing body of evidence suggests that the Fe(II)/α-ketoglutarate-dependent oxygenase AlkB homolog 5(ALKBH5) demethylates RNA N6-methyladenosine (m^6^A) [148–150]. Zhang et al. indicated that ALKBH5 was upregulated in glioblastoma stem cells, demethylated FOXM1 nascent transcripts, upregulated FOXM1, and promoted cell cycle progression [151]. ALKBH5 upregulation is also required for AML progression and leukemia stem cell self-renewal [152, 153]. Conversely, ALKBH5 repression decreased tumorsphere formation and downregulated the TFs SOX2, Nanog, and Oct4, contributing to the self-renewal capacity of glioblastoma stem cells and breast CSCs [154, 155]. These findings collectively suggest that changes in the intracellular LIP affect the activity of the previously mentioned enzymes, mediate downstream epigenetic alterations, and influence CSCs biology.

## Targeting treatments that modulate CSCs iron metabolism

**Fig. 3:**
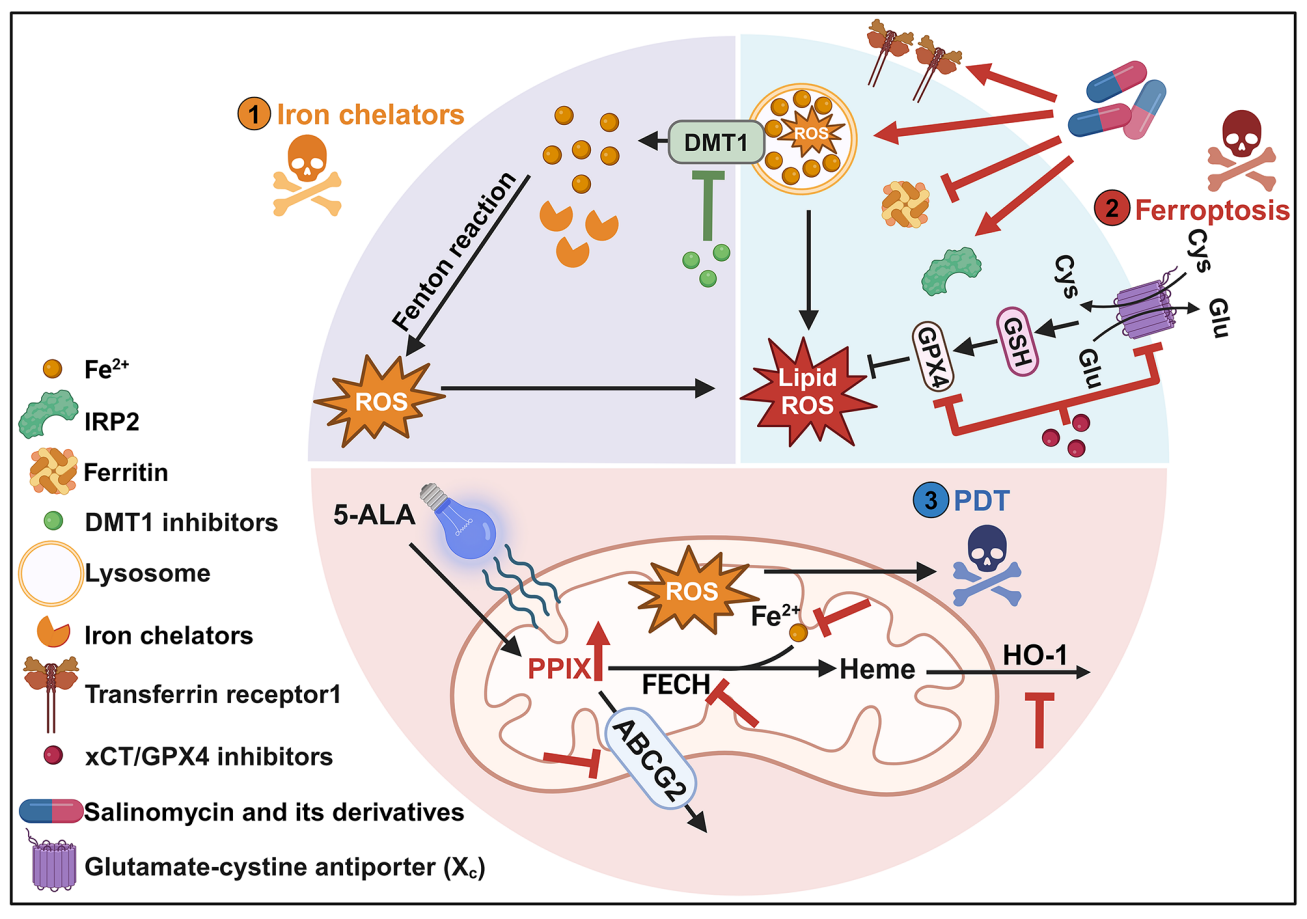
Current therapeutic strategies that eradicate cancer by targeting CSCs. (1) Iron chelators. (2) Ferroptosis-based drugs. (3) 5-ALA-based PDD/PDT. **Abbreviations**: ABCG2, ATP-binding cassette sub-family G member 2; 5-ALA, 5-aminolevulinic acid; PDD, photodynamic diagnosis; PDT, photodynamic therapy; PPIX, protoporphyrin IX; HO-1, heme oxygenase-1; FECH, ferrochelatase; DMT1, divalent metal transporter 1; GPX4, glutathione peroxidase 4; GSH, glutathione; Cys, cystine; Glu, glutamate; ROS, reactive oxygen species; TFR1, transferrin receptor 1; IRP2, iron regulatory protein 2

To address the iron addiction of CSCs, two main strategies have been reported: (1) disrupt CSCs biology by reducing their iron content with iron chelators and (2) induce ferroptosis, a new form of cell death triggered by a combination of iron toxicity, build-up of lipid peroxides on cellular membranes. The relevant compounds are summarized in Table 1. Additionally, novel treatments targeting iron metabolism, such as PDT and PDD, hold promise for targeting CSCs (Fig. 3).

**Table 1:** Overview of the latest compounds targeting iron metabolism for CSCs therapy

	Drug	Mechanism	Tumor Type	References
Iron chelators	Deferoxamine (DFO)	Chelating iron	Lung cancer, Ovarian cancer, Leukemia, Breast cancer	[112, 156–158]
	Deferasirox (DFX)	Chelating iron	Lung cancer, Leukemia, Squamous cell carcinoma, Esophageal adenocarcinoma	[156, 157, 159]
	Di-2-pyridylketone-4,4-dimethyl-3-thiosemicarbazone (Dp44mT)	Chelating iron	Medulloblastoma, Breast cancer	[158, 160]
Ferroptosis-based drugs and nanoparticles	Salinomycin, Ironomycin, Phenazine derivatives, Itraconazole, Dichloroacetate	Sequestering iron in lysosomes	Breast cancer, Nasopharyngeal carcinoma, Colorectal cancer	[175–177, 199]
	Ebselen, Substituted pyrazoles, Benzylisothioureas	DMT1 inhibitors	Breast cancer	[178]
	Temozolomide & quinacrine	Reducing the activity of GPx and accumulating lipid peroxides	Glioblastoma	[179]
	RSL3	Targeting and inhibiting GPX4	Ovarian cancer	[174]
	Erastin, Sulfasalazine, Epothilone analogs, C2-4, Open chain epothilone analogues, Gallocyanine	Inhibition of system Xc-	Ovarian cancer, Colorectal cancer, Lung cancer Gastric cancer, Breast cancer, Neuroblastoma	[107, 168, 173, 200–203]
	GNPIPP12MA	Suppressing FTO/m6A methylation through depleting GSH	Acute myeloid leukemia	[204]
	FeOOH/siPROM2@HA	Inhibiting Fe^3+^ efflux to promote its redox reaction with endogenous GSH to produce Fe^2+^ and initiate the Fenton reaction-based ferroptosis by lipid peroxidation elevation	Breast cancer	[205]
	AuNP-PHF	Inducing ferritin degradation	Breast cancer	[206]
	Atranorin@SPION	Inhibiting Xc- and GPX4	Gastric cancer	[180]
	Sal-AuNPs	Iron accumulation and inhibition of antioxidant properties	Breast cancer	[207]
	RF@LA-Fe-MOF	Releasing RSL3 and the inhibitor of FSP1(ferroptosis suppressor protein 1) to inhibit the levels of GPX4 and FSP1 and increase lipid peroxides	Hepatocellular carcinoma	[208]

### Iron chelators

Given that CSCs have high Fe demand, it is highly conceivable that iron chelating agents could eradicate them. Common Fe chelating agents include DFO, deferasirox (DFX), and Dp44mT [112, 156–160]. However, they have short circulatory half-lives and do not target specific tumors. Thus, they have been evaluated in preclinical research but have not yet been approved for clinical applications. Furthermore, the substantial iron buffering capacity of cancer stem cells significantly restricts the therapeutic efficacy of iron chelators. Induced iron deficiency by these chelators leads to the accumulation of HIF-1α [161, 162], which maintains CSCs stemness under hypoxic conditions through activation of self-renewal signaling pathways, including Notch, Wnt, and Hedgehog pathways. Additionally, HIF-1α promotes chemoresistance by mediating drug efflux through transporters like P-glycoprotein (ABCB1) and ABCG2, and by inducing epithelial-mesenchymal transition (EMT) in CSCs [163–166].

### Ferroptosis

Iron and high reactive oxygen species levels present a double-edged sword in CSCs. While essential for biological processes, excess iron fuels the Fenton reaction, generating abundant ROS, disrupting intracellular redox homeostasis, oxidizing polyunsaturated fatty acids (PUFAs), triggering lipid peroxidation, and ultimately inducing ferroptosis [167]. This vulnerability makes ferroptosis a promising avenue for eradicating CSCs. Studies have shown high concentrations of the CD44 variant (CD44v) in CSCs, where it interacts with and stabilizes the glutamate-cystine transporter xCT. This promotes the uptake of cystine, which is vital for producing the ferroptosis repressors reduced glutathione (GSH) and glutathione peroxidase 4 (GPX4) [168–170]. Therefore, inhibiting the xCT/GSH/GPX4 pathway holds significant potential for eliminating CSCs. Erastin, sulfasalazine (SASP), epothilone analogs, etc. are promising xCT inhibitors demonstrating therapeutic potential in anti-CSCs therapy [171–174].

Salinomycin and its synthetic derivative ironomycin offer new approaches to eliminate CSCs. They induce cytoplasmic iron depletion, increasing the levels of IRP2 and TFR1, promoting lysosomal degradation of ferritin, sequestering iron in lysosomes, amplifying iron-mediated lysosomal ROS production, and ultimately driving CSCs ferroptosis [175]. Similarly, repurposing old drugs like phenazine derivatives and itraconazole has shown efficacy in inducing ferroptosis in breast and nasopharyngeal CSCs through mechanisms essentially identical to those of salinomycin and ironomycin [176, 177]. Finally, inhibiting the divalent metal transporter 1 (DMT1) using ebselen, substituted pyrazoles, or benzylisothioureas blocks lysosomal iron transport, leading to lysosomal iron accumulation, ROS production, and ferroptosis in breast CSCs [178]. In addition, Temozolomide & quinacrine could reduce the activity of glutathione peroxidase (GPx) and promote the accumulation of lipid radicals to induce ferroptosis [179]. Nanoparticles have also been reported to be involved in the induction of ferroptosis in CSCs, such as Atranorin@SPION, a complex of superparamagnetic iron oxide nanoparticles (SPION) and Atranorin, could attenuate the mRNA 5-hydroxymethylcytidine modification of the cystine/glutamate transporter (Xc-)/glutathione peroxidase 4 (GPX4) axis and its expression in gastric CSCs [180].

### PDD/PDT

The amino acid 5-aminolevulinic acid (5-ALA) and its downstream product, protoporphyrin IX (PpIX), are not only precursors in heme biosynthesis but also serve as classical photosensitizers. Photodynamic diagnosis (PDD) and photodynamic therapy (PDT) harness the photon-induced physicochemical reactions of these photosensitizers, showing promise as anticancer therapies [181, 182]. Upon exposure to light, photosensitizers generate reactive oxygen species in abundance, triggering apoptosis, vascular damage, and immunogenic cell death (ICD) [183]. However, in the presence of iron ions, the enzyme FECH can catalyze the conversion of PpIX to heme, leading to a loss of its photosensitivity and reduced PDT efficacy [184, 185]. Interestingly, studies have shown that PDD and PDT based on 5-ALA could enhance the radiosensitivity of prostate cancer by downregulating stem cell markers and inhibiting sphere formation [186].

Wang et al. proposed that the downregulation of 5-ALA-derived PpIX and the upregulation of HO-1 accelerate PpIX-heme metabolism and contribute to PDT resistance in glioblastoma CSCs [187]. To enhance PDT’s effectiveness in combating these CSCs, several strategies can be employed. Firstly, PPIX excretion could be reduced by inhibiting ABCG2, a potential stem cell marker linked to low PDD/PDT efficiency [188, 189]. Secondly, PPIX utilization can be hindered. Since ferrous iron incorporation by ferrochelatase metabolizes PPIX to heme, reducing FECH activity [190] or eliminating ferrous iron [187, 191] could restore intracellular PPIX levels. Additionally, uninterrupted reactive oxygen species generators (URGs) could be utilized to induce sustained ROS production. By converting heme into peroxidase mimics, URGs enable the subsequent conversion of H_2_O_2_ to hydroxyl radicals (•OH), leading to sequential mitochondrial and nuclear damage, potentially proving highly toxic to CSCs [192].

## Conclusions and future perspectives

Extensive researches have demonstrated that iron, a crucial micronutrient metal in the human body, is present in abundance across various cell types and tissues, fulfilling diverse and distinct biochemical and physiological functions. Notably, accumulating evidence from literature reviews suggests a strong correlation between iron levels and cancer progression. Given the significant role of cancer stem cells in tumor recurrence, metastasis, and resistance to therapy, the current review investigates the functions of iron within these cell populations. Importantly, we sought to provide a concise overview of iron’s pivotal roles in CSCs metabolism, epigenetic processes, and epithelial-mesenchymal transition, highlighting their potential contribution to the development of novel therapeutic strategies targeting CSCs. Promising anti-CSCs approaches explored include ferroptosis-based drugs, 5-ALA-PDT, and iron chelators. However, the significant toxicity and severe side effects associated with iron chelators raise concerns regarding their clinical applicability. Interestingly, nanotechnology-based drug delivery systems offer a potentially avenue to enhance the feasibility and practicality of using iron chelators as an anti-CSCs therapy, as demonstrated in previous studies [193].

Leveraging the diverse biological activities of nano-drug encapsulation and delivery technologies, researchers have explored them as potential anticancer treatments. Given the dependence of CSCs on iron metabolism, innovative strategies could involve iron chelator-based nanostructures and nanotherapeutic drugs that induce iron accumulation and subsequent generation of toxic ROS within CSCs. Lang et al. identified TFR1-targeting liposomes for co-delivering DFO and the HIF-1α inhibitor lificiguat (YC-1). Following intravenous injection in mice, the nanoparticles passively accumulated in tumor xenografts via the enhanced permeability and retention (EPR) effect [194]. Subsequently, the encapsulated DFO and YC-1 were released. Nanoparticulate DFOs (TNP-DFO-YC-1) exhibited significantly extended circulation times compared to free DFO. Moreover, YC-1 significantly enhanced the inhibitory effect of DFO against pancreatic CSCs by impeding tumor spheroid formation [195]. Encapsulation of Dp44mT in poly (lactic-co-glycolic acid) (PLGA) NPs demonstrably hindered spheroid growth and promoted apoptosis [196]. Additionally, paramagnetic, blood-compatible Fe_3_O_4_ NPs and SnFe_2_O_4_ nanocrystals served as Fenton reaction catalysts, converting H_2_O_2_ into cytotoxic hydroxyl radicals (•OH), thereby inducing apoptosis in glioblastoma, breast, and colorectal cancers [197, 198]. These findings suggest their high potential for eradicating CSCs as well. In conclusion, nanocarriers represent promising therapeutic avenues for targeting and eliminating CSCs.

## Data Availability

No datasets were generated or analysed during the current study.
